# Correction: Click activated protodrugs against cancer increase the therapeutic potential of chemotherapy through local capture and activation

**DOI:** 10.1039/d1sc90098f

**Published:** 2021-05-21

**Authors:** Kui Wu, Nathan A. Yee, Sangeetha Srinivasan, Amir Mahmoodi, Michael Zakharian, Jose M. Mejia Oneto, Maksim Royzen

**Affiliations:** University at Albany, SUNY 1400 Washington Ave., LS-1136 Albany NY 12222 USA mroyzen@albany.edu; Shasqi, Inc. 665 3^rd^ St., Suite 501 San Francisco CA 94107 USA jose@shasqi.com

## Abstract

Correction for ‘Click activated protodrugs against cancer increase the therapeutic potential of chemotherapy through local capture and activation’ by Kui Wu *et al.*, *Chem. Sci.*, 2021, **12**, 1259–1271, DOI: 10.1039/D0SC06099B.

The authors regret that the reference to the bond-breaking bioorthogonal chemistry, termed ‘click-to-release’ was omitted from the original article. In addition, we would like to include a reference describing the synthesis of compound **1**, which is an intermediate to the protodrugs described in the original article. These references are listed below as ref. 1 and 2.

The Royal Society of Chemistry apologizes for these errors and any consequent inconvenience to authors and readers.
